# Two-dimensional material-based bionano platforms to control mesenchymal stem cell differentiation

**DOI:** 10.1186/s40824-018-0120-3

**Published:** 2018-04-02

**Authors:** Ee-Seul Kang, Da-Seul Kim, Intan Rosalina Suhito, Wanhee Lee, Inbeom Song, Tae-Hyung Kim

**Affiliations:** 1 School of Integrative Engineering, Chung-Ang University, 84 Heukseok-ro, Dongjak-gu, Seoul, 06974 Republic of Korea; 2 Integrative Research Center for Two-Dimensional Functional Materials, Institute of Interdisciplinary Convergence Research, Chung-Ang University, Seoul, 06974 Republic of Korea

**Keywords:** Graphene, Two-dimensional materials, Gold nanoparticles, Three-dimensional graphene composites, Human mesenchymal stem cell, Differentiation

## Abstract

**Background:**

In the past decade, stem cells, with their ability to differentiate into various types of cells, have been proven to be resourceful in regenerative medicine and tissue engineering. Despite the ability to repair damaged parts of organs and tissues, the use of stem cells still entails several limitations, such as low differentiation efficiency and difficulties in guiding differentiation. To address these limitations, nanotechnology approaches have been recently implemented in stem cell research. It has been discovered that stem cells, in combination with carbon-based functional materials, show enhanced regenerative performances in varying biophysical conditions. In particular, several studies have reported solutions to the conventional quandaries in biomedical engineering, using synergetic effects of nanohybrid materials, as well as further development of technologies to recover from diverse health conditions such as bone fracture and strokes.

**Main text:**

In this review, we discuss several prior studies regarding the application of various nanomaterials in controlling the behavior of stem cells. We focus on the potential of different types of nanomaterials, such as two-dimensional materials, gold nanoparticles, and three-dimensional nanohybrid composites, to control the differentiation of human mesenchymal stem cells (hMSCs). These materials have been found to affect stem cell functions via the adsorption of growth/differentiation factors on the surfaces of nanomaterials and the activation of signaling pathways that are mostly related to cell adhesion and differentiation (e.g., FAK, Smad, Erk, and Wnt).

**Conclusion:**

Controlling stem cell differentiation using biophysical factors, especially the use of nanohybrid materials to functionalize underlying substrates wherein the cells attach and grow, is a promising strategy to achieve cells of interest in a highly efficient manner. We hope that this review will facilitate the use of other types of newly discovered and/or synthesized nanomaterials (e.g., metal transition dichalcogenides, non-toxic quantum dots, and metal oxide frameworks) for stem cell-based regenerative therapies.

## Background

Recently, a wide variety of stem cells have been investigated for their extensive utility in biomedical applications, owing to their abilities to differentiate into specific cell lineages, and to generate more stem cells. Mesenchymal stem cells (MSCs), which are multipotent stromal stem cells, have been extensively investigated for their accessibility, versatility, and low risk of teratoma formation. Their multipotency allows them to differentiate into several specific cell types (e.g. adipocytes, osteoblasts, chondrocytes), to form fat [1], bone [2], and cartilage tissues [3]. Traditionally, the process of stem cell differentiation has been controlled using media containing specific regulator proteins and biomolecules (e.g., dexamethasone, ascorbic acid, and β-glycerophosphate) [4]. However, since cells actively interact with the underlying substrates/surfaces wherein they attach and grow, a method for controlling their functions including proliferation, migration, and differentiation, via biophysical factors, instead of induction media or the combination of two, has been recently proposed [5–7]. Such biophysical stimuli are induced by modifying the substrate/surface with cell-matrix interactions, which ultimately influence both cytoskeletal mechanics and cellular gene/protein expression [8, 9].

Until now, a variety of nanomaterials, including carbon nanotube (CNT) [10], fullerenes, and graphene [11], have been reported to guide stem cell differentiation with or without the presence of soluble differentiation factors. Among such materials, graphene and its derivative, graphene oxide (GO), have gained attention as unique materials to induce the physical stimulation required for stem cell differentiation. It has been reported that these features of amphiphilicity, surface chemistry, and honeycomb structures of GO [12, 13] affect cytoskeletal dynamics of cells adhered to the GO surface, which ultimately result in the changes of cell spreading, morphology and proliferation [14–16].

Several studies have reported the application of two-dimensional materials including graphene and its derivatives for productive differentiation of stem cells into desired lineages. Particularly, graphene oxide micropatterns, graphene nanopatterns, graphene, and nanomaterials hybrid platforms have been reported to promote the differentiation of hMSCs into osteocytes, adipocytes, and chondrocytes [17–22]. This is also attributed to unique surface properties such as absorption/repulsion of specific differentiation factors, and the enhancement of cell adhesion through interactions between the cell membrane and the surface of the carbon materials [23, 24].

**Fig. 1:**
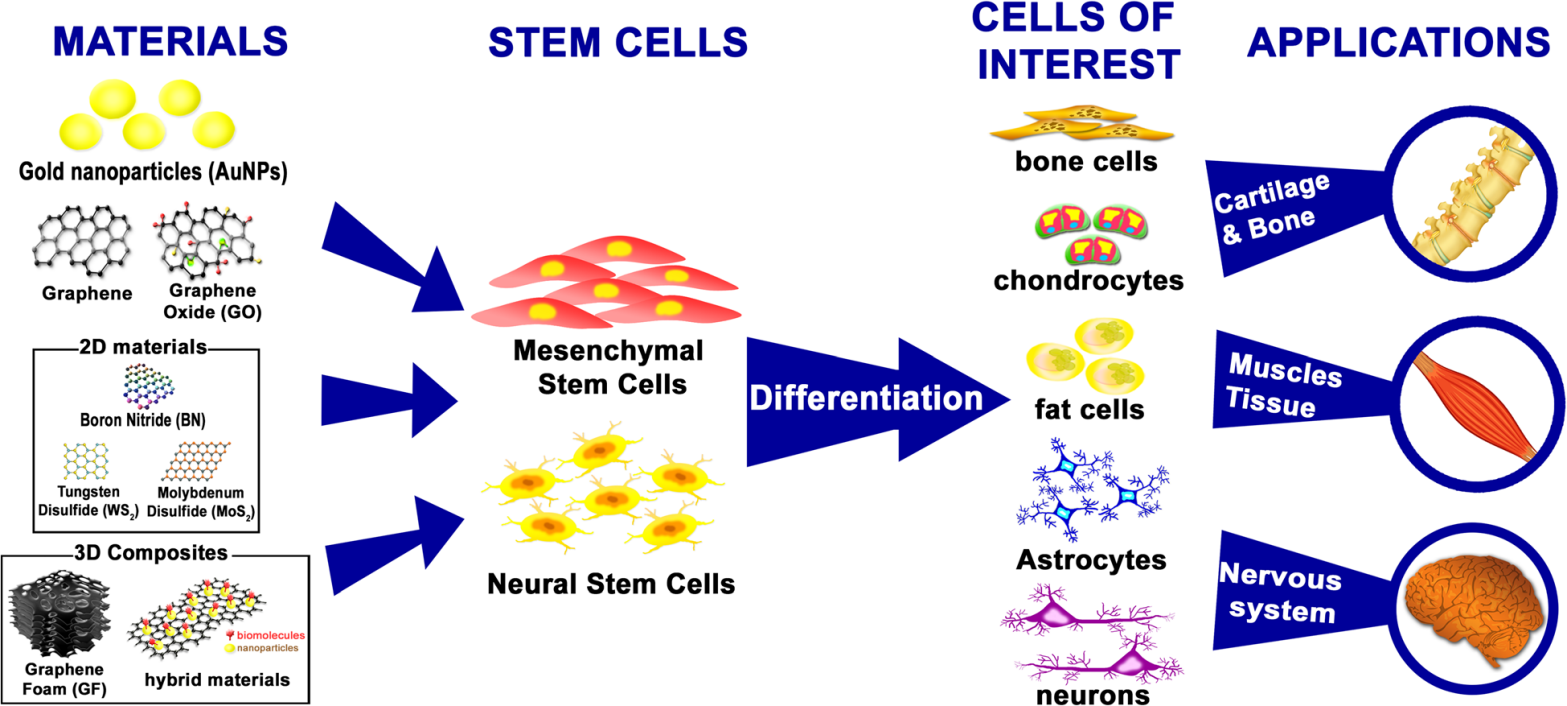
Schematic illustration of stem cells differentiation using bionano hybrid materials and their applications

On the other hand, other nanomaterials have been tested for drug delivery and other stem cell therapeutic applications [25–27]. Their particle size, large surface area, and an ability to translocate into cells have shown promising prospects in noble biomedical utilizations [28–30]. Gold nanoparticles (AuNPs), a representative material in biomedical research, are best known for relatively low cytotoxicity, biocompatibility, and versatility on surface modification [31–33]. Moreover, efforts have been made to apply three-dimensional structures to cell culture experiments [34–36]. Henceforth, this study focuses on the following three categories: (i) controlling the hMSCs using carbon-based materials, (ii) differentiation of hMSCs through nanomaterials, and (iii) effects of bionano platform on cell behaviors. We have narrowed our focus to bionano hybrid platform to two-dimensional materials, AuNPs, RGD peptide (arginyl-glycyl-aspartic acid), and silica nanoparticles, all of which are known to be good for cell adhesion. (Fig. 1).

## Main text

### Mesenchymal stem cells behaviors on two-dimensional materials

hMSCs have been shown as a promising source for stem cell therapies and regenerative medicine due to their ability to self-renew and differentiate toward various types of cells, such as osteocytes, adipocytes, and chondrocytes [37–40]. In addition, they can be easily isolated from the bone marrow, fat, and umbilical cord, and successfully expanded in vitro [41, 42]. However, several carbon-based materials have been lauded as versatile tools for establishing the future generation of biomaterials [43–45]. Although each carbon-based material, such as fullerene, carbon nanotubes, and graphene, presents its own advantages and disadvantages, graphene and its derivatives in particular have been used to guide the behavior of hMSCs [21, 46–48]. Graphene has several features that are advantageous for biomedical applications, owing to unique physiochemical properties, from its surface chemistry, amphiphilicity, and specific carbon structures [49]. Subsequently, graphene and GO, once fully exploited, would drastically influence the spreading, morphology, and proliferation of stem cells, and become prospects for osteogenic differentiation of hMSCs [50, 51].

**Fig. 2:**
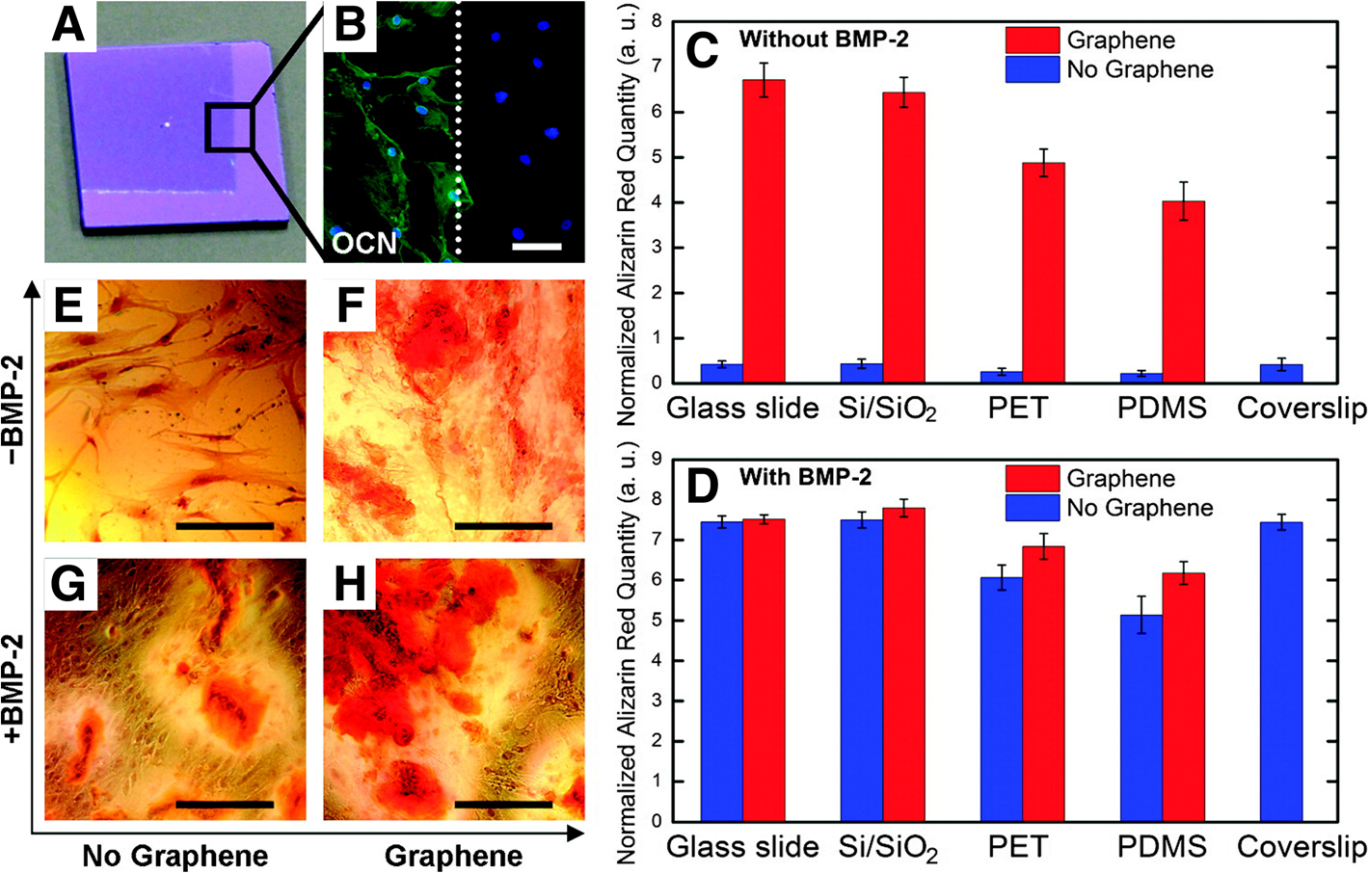
Enhancement of osteogenic differentiation on graphene substrates with/without BMP-2. (**a**) Optical image of graphene-coated Si/SiO_2_ substrate. The boundary is shown for the graphene-coated part. (**b**) Osteocalcin (OCN) staining, a marker of osteogenic differentiation. Green = OCN, Blue = DAPI. (**c**, **d**) Alizarin Red S (ARS) quantification graphs during 15 days on substrates with/without graphene. (**e**-**h**) polyethylene terephthalate (PET) substrate stained with ARS, showing calcium deposits due to osteogenic differentiation. Reprinted with permission from [11]. Copyright (2011) American Chemical Society

Generally, carbon-based materials are prepared by chemical vapor deposition (CVD), which ensures high quality and high volume production, before being transferred to a variety of substrates [24, 52]. For instance, graphene is usually functionalized in order to enhance the bioactivity of hybrid composite before being used as a surface coating on biomaterial substrates [53]. Many researches have reported that graphene has the ability to guide osteogenic differentiation of hMSCs. For example, Nayak et al. found that graphene induces osteogenic differentiation when cultured without BMP-2, a common growth factor in bone formation [11] (Fig. 2). The stark difference in alizarin red s (ARS) data between Fig. 2b and c show that calcification in graphene is higher even in the absence of BMP-2, and Fig. 2e-h show that osteogenesis differs depending on the presence or absence of graphene and BMP-2.

On the other hand, Lee et al. [4] discovered that the binding ability of graphene with several osteogenic differentiation factors could enhance the differentiation of hMSCs into the osteogenic lineage. They conducted an experiment wherein they cultured hMSCs on the CVD graphene. Several osteogenic differentiation factors, such as dexamethasone, ascorbic acid, and β-glycerophosphate, were used in the culturing process. The result showed that graphene had the ability to promote osteogenesis of hMSCs within 12 days, which was 9 days shorter than the prior studies. It indicates that osteogenesis in the presence of graphene could be achieved earlier than with conventional substrates.

**Fig. 3:**
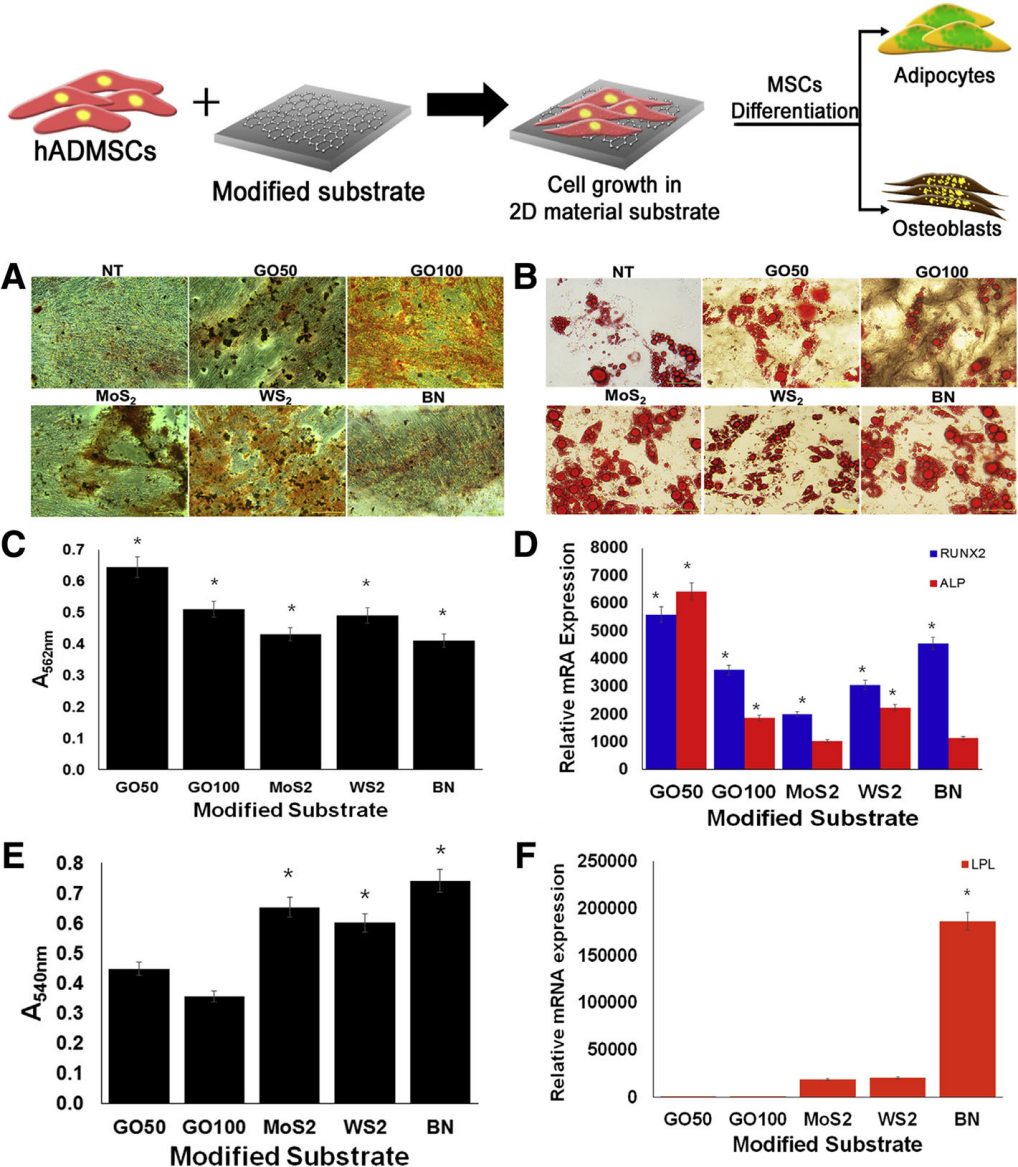
The various differentiation indicators in accordance with their respective 2D materials substrates. (**a**) Optical images of osteogenesis confirmed by ARS. (**b**) Oil Red O (ORO) staining images of each substrates. (**c**) Quantification graph of ARS result, and (**d**) gene expression level of osteogenic markers by qPCR. (**e**) ORO results converted to a quantified graph. (**f**) qPCR analysis data on adipogenic marker gene of expression level. (* *p* < 0.05) (GO: graphene oxide, MoS2: molybdenum sulfide, WS2: tungsten sulfide, BN: boron nitride) Copyright 2017, Royal Society of Chemistry

In addition, Suhito et al. compared the osteogenic differentiation of hMSCs on graphene oxide and other graphene-like 2D materials such as molybdenum sulfide (MoS_2_), tungsten sulfide (WS_2_), and boron nitride (BN) [54]. Figure 3 visualizes osteogenic and adipogenic differentiation in hMSCs using the 2D materials mentioned above. As shown in Fig. 3 (a), (c), and (d), the osteogenic differentiation was confirmed on each substrate, and most of the hMSCs grown on each substrate were fully differentiated. However, the results from optical microscopy, ARS, and qPCR showed that the best differentiation rate was obtained at the GO concentration of 50 μg/mL.

Figure 3 (b) and (e), represent the oil red O (ORO) staining, which stains lipids when hMSCs are differentiated into adipocytes, demonstrating the adipogenetic capacity of hMSCs. Upon quantification, it was confirmed that the rate of adipogenesis in other substances was much higher than that in GO. Moreover, Fig. 3 (f) shows that the gene expression level of the hMSCs grown on BN substrates was the highest among the test substrates.

In addition, it is also known that graphene oxide nanoribbon (GONR) and reduced graphene oxide nanoribbon (rGONR) grids influence the osteogenic differentiation and proliferation of hMSCs, regardless of the presence or absence of differentiation inducing factors [18].

When osteogenic factors were present, the fastest osteogenic differentiation of hMSCs in rGONR grids was found to occur in about 7 days. The rapid osteogenic differentiation in rGONR was thought to be due to the high adsorption of differentiation inducing substances by rGONR and the physical properties induced by the surface characteristics of the nanogrids.

Moreover, many studies have confirmed the effects of graphene on the differentiation of other stem cells as well as hMSCs. For example, Chen et al. [55] reported the biocompatibility of G and GO toward long-term culture of induced pluripotent stem cells (iPSCs). Interestingly, iPSCs cultured on G and GO showed imbalance in differentiation tendencies. Especially, in the endodermal lineages, G interrupted spontaneous differentiation. On the other hand, GO promotes the differentiation of iPSCs most prominently along the ectodermal pathway, but differentiation into ectoderm and mesodermal is similar to iPSC incubated in both G and GO.

Consequently, it was found that graphene, GO, and other two-dimensional materials with their unique chemical and physical characteristics, enhance and guide the osteogenic or adipogenic differentiation of hADMSCs. In addition, we could confirm that 2D materials have various effects on the differentiation of hMSCs as well as other types of stem cells. This demonstrated that carbon-based materials were potential materials not only for regenerative medicine but also for the biomedical fields.

### The effect of gold nanoparticles (AuNPs) on hMSCs growth and differentiation

Another type of nanomaterial with broad potential in biomedical application is gold nanoparticles (AuNPs). As mentioned already, AuNPs have been proposed as an attractive material for regenerative medicine, owing to their favorable physical properties, including biocompatibility arising from their low cytotoxicity, and abundant control over the particle size [56–58]. Numerous studies have investigated their application in biomedical fields such as biological imaging, chemical sensing, drug carriers, and disease treatments [59–63]. Most importantly, the negative charge on the surface of AuNPs makes gold nanoparticles more easily modifiable than the other NPs, such that the AuNPs can be functionalized by a wide range of biomolecules, drugs, DNA, antibodies, and functional peptides/polymers for favorable biomedical research and therapy [64].

Previously, AuNPs, functionalized with polymers such as chitosan-conjugated AuNPs, were developed to achieve advanced differentiation of human mesenchymal Stem Cells (hMSCs) [65]. Chitosan, a type of aminated polysaccharide that has been utilized in bone tissue engineering, shows similarity to glycosaminoglycan, which plays an important role in extracellular matrix (ECM) interaction during cell adhesion. Moreover, further investigation discovered that chitosan polymers can promote osteogenic differentiation through Wnt/β-catenin signaling pathway [66, 67]. However, AuNPs themselves have been found to promote osteogenic differentiation of hMSCs by their stimulation through protein kinase 38 (p38) mitogen-activated protein kinase (MAPK) pathway. The difference in charge and the moiety of AuNPs have been shown to induce a series of cell responses towards osteogenesis [33]. Hence, Yi et al. studied the use of AuNPs as a novel biomaterial to enhance the osteogenic differentiation of hMSCs and the associated molecular mechanisms [33].

**Fig. 4:**
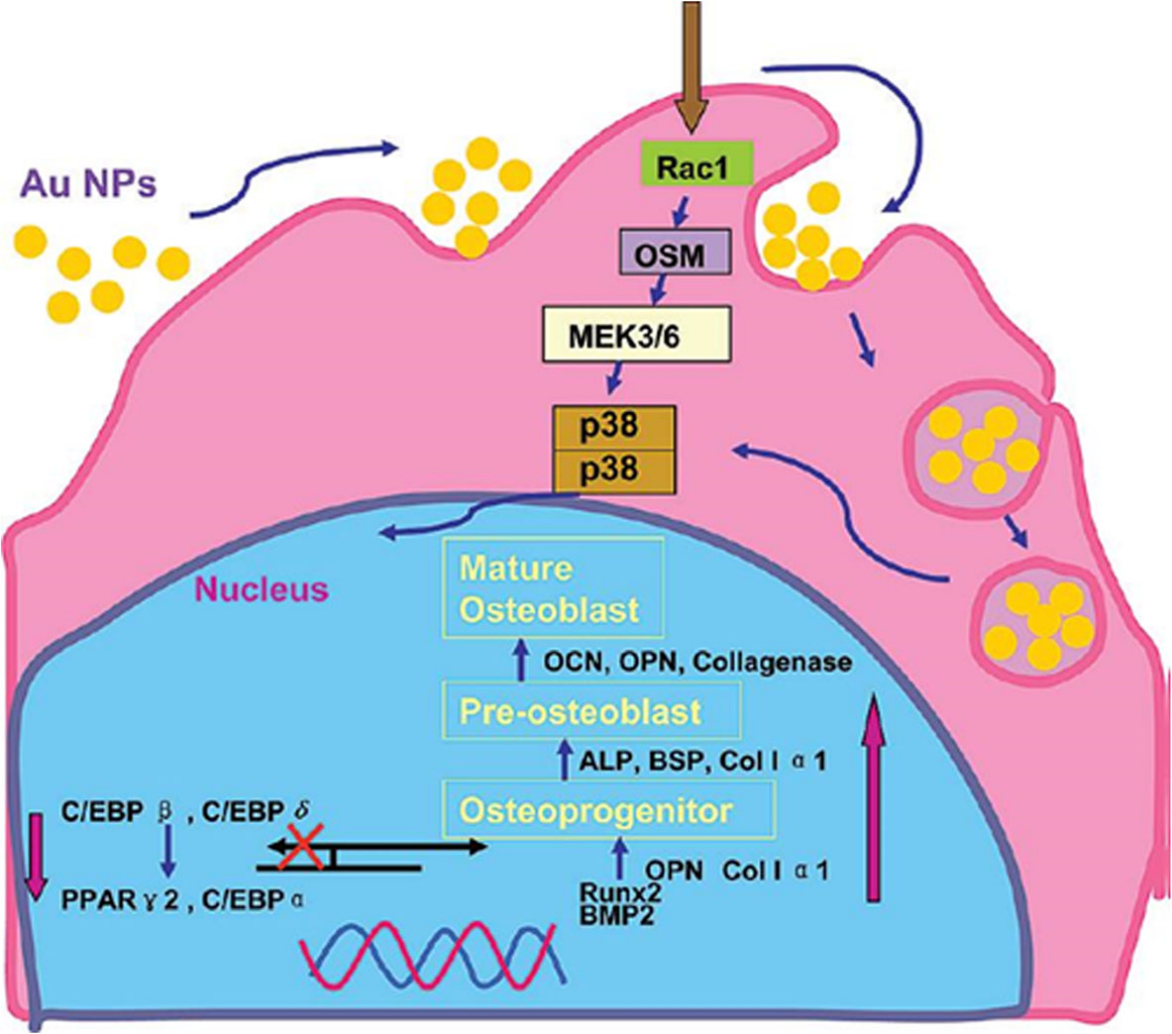
Illustration showing molecular mechanism of the modulation of osteogenic differentiation of hMSCs by AuNPs through p38 MAPK signaling pathway. Reprinted with permission from [29]. Copyright 2017 American Chemical Society

Figure 4 illustrates the role of AuNPs in terms of gene regulation through osteogenesis of hMSCs. The AuNPs would attach to the hMSC membrane and bind to proteins in the cytoplasm. This is followed by internalization via endocytosis, which induces mechanical stress in the cell. It has been revealed that several signaling molecules play an important role in signal transduction. A hypothesis stated that AuNPs may serve as mechanical stimulator for hMSCs in terms of the activation of MAPK signaling pathway in the cells, thus, inducing their preferential differentiation. The stimulation of p38 MAPK signaling mechanism leads to an up-regulation of transcription factors that are related to osteogenic differentiation, such as RUNX2. It then subsequently triggers several marker genes for osteogenesis, such as Col I and BMP-2 at the early stages, and ALP and OCN at the later stages of differentiation. According to other type of hMSC differentiation such as adipogenesis, the activation of p38 MAPK pathway delivers to the down-regulation of adipogenic marker genes, e.g., PPARγ and C/EBPα [68, 69]. Therefore, AuNPs could inhibit the adipogenic differentiation of hMSCs.

**Fig. 5:**
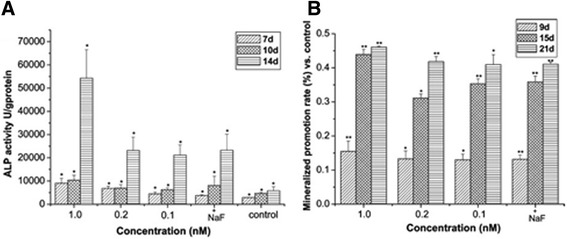
The effects of gold nanoparticles (AuNPs) on osteogenesis of human mesenchymal stem cells (hMSCs). (**a**) Effects of AuNPs on the ALP activity of hMSCs. Results are mean ± SD of triplicate experiments: (*) *p* < 0.01. (**b**) Effects of AuNPs on the mineralized nodule formation of hMSCs. Mineralization quantitated by elution of Alizarin Red S from stained mineral deposits. Results are mean ± SD of triplicate experiments: (*) *p* < 0.05, (**) *p* < 0.01. NaF at 1 μM used as a positive control for both experimental data. Reprinted with permission from [29]. Copyright 2017 American Chemical Society

Based on the results shown in Fig. 5a, the assessment of ALP activity from hMSCs cultured on 7, 10, and 14 days effectively demonstrates the effect of AuNPs toward osteogenesis. These data represent the increase in ALP activity due to stronger promotion of osteogenic differentiation of hMSCs followed by the increase in AuNP concentration, especially on day 14. In addition, ARS staining assay was performed to account for the mineralization in osteoblasts. In Fig. 5b, the AuNPs showed similar promotive effects on mineral formation in hMSCs. The ARS quantitative data referring to the mineralized-nodules in osteoblasts upon AuNPs treatment was significantly increased in a dose- and time-dependent manner. At day 21, the mineralization in the presence of 1 nM AuNPs was 45% higher than those of other groups (see Fig. 5b) [33].

Therefore, it can be concluded that AuNP surface functionalization with biomolecules is an effective strategy to enhance stem cell growth and differentiation. Although the use of AuNPs is highly promising in the field of stem cells for regenerative and therapy, further studies are needed to examine and develop the compatibility of various molecules in terms of nanoparticles conjugation for biological research.

### Controlling differentiation of hMSCs using modified 3D graphene-based platform

**Fig. 6:**
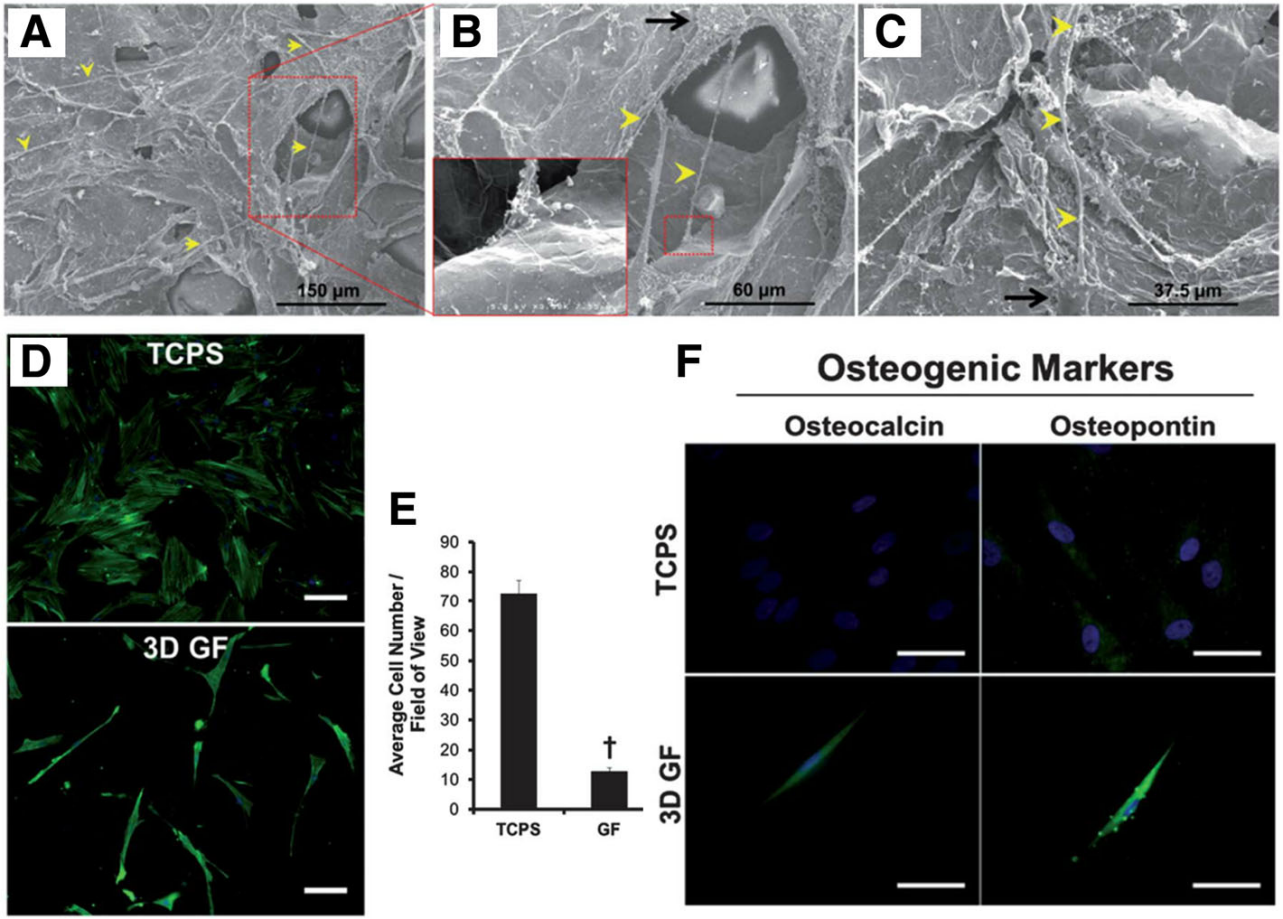
The effects of 3D graphene foams (GFs) on the adhesion and osteogenic differentiation of human mesenchymal stem cells (hMSCs). (**a** - **c**) The SEM images of hMSCs cultured on 3D GFs for 4 days. The yellow arrows represent formed protrusions up to 100 mm in length that extended from small cell bodies (black arrows). (**d**) Immunofluorescence images of hMSCs cultured on TCPS and 3D GFs for 7 days. (**e**) The average cell number was quantified from Fig. 6d. (**f**) Immunofluorescence images stained with osteogenic markers, Osteocalcin and Osteopontin, for hMSCs cultured on TCPS and GF for 7 days. Scale bar = 50 μm. Copyright © 2013, Royal Society of Chemistry

In terms of controlling the differentiation of hMSCs, various attempts have been made using a modified platform. Especially, a platform that modified three-dimensional (3D) graphene-based substrates has been currently in spotlight due to its similarity with the 3D microenvironment ECM in human body [70–75]. It has been shown that the transport behaviors of cytokines, chemokines, and growth factors are significantly different in 2D and 3D microenvironments, which would consequently influence signaling transduction, cell-cell communications, and tissue development [9, 76–80]. To address these issues, we highlighted the various developments that have been made to differentiate the hMSCs with graphene-based 3D platform in relation to notable properties of graphene mentioned above (see Table 1). First of all, 3D graphene foams (GFs) were utilized as an hMSCs cultivation substrate [81]. It is known that 2D graphene sheets can accelerate the differentiation of hMSCs in presence of osteogenesis induction media [11, 24]. Crowder et al. hypothesized that a 3D GF would accelerate differentiation of hMSCs more effectively than a 2D graphene sheet. Based on previous studies, 3D GFs have been utilized for multifarious applications such as battery technology and electrochemical sensing [82, 83]. However, the authors investigated that 3D GFs are capable of being used as novel culture substrates for cell growth and inducing spontaneous osteogenic differentiation of hMSCs. Figure 6a-c show SEM images of hMSCs cultured on GFs for 4 days. Interestingly, the protrusion of the cells spread across large pores in the GF and interact with the GF surface. We discovered that the 3D GFs were highly porous, with individual pore sizes exceeding 100 mm, and that the hMSCs had sensed and spanned across the pores. In Fig. 6d, hMSCs morphology seems significantly different on GFs compared with the tissue culture polystyrene (TCPS). As the GFs have a highly porous 3D structure, as shown in Fig. 6e, the attachment of hMSCs cultured in GF was observed to be much lower than that on TCPS. However, the cells were spontaneously stimulated into osteogenic differentiation (Fig. 6f), even though the cell culture media did not contain osteogenetic inducers. In addition, due to the physical properties of GFs, such as flexibility and conductivity [84, 85], GFs have been studied for effective proliferation and differentiation of human neural stem cells (hNSCs) in the presence of electrical stimulation [86]. Akhavan et al. discovered that hNSCs, grown on the GF with electrical stimulation, resulted in a much higher rate of proliferation and accelerated differentiation into neurons.

**Table 1:** Summary of studies using 3D graphene-based substrate for differentiation of MSCs

3D platform	Type of carbon-based material	Type of cells	Outcomes	Reference
GFs	Graphene foam	hBMMSCs	GFs promoted osteogenic differentiation without the use of chemical inducers.	[81]
rGO-Collagen hybrid (PADM-rGO)	Reduced Graphene Oxide	rBMMSCs	PADM-rGO promoted the differentiation of MSCs into neural cells after 7 days under neural differentiation condition.	[102]
GC	Graphene	hBMMSCs	The graphene / calcium silicate (GC) composite coating promoted adhesion and osteogenic differentiation of hMSCs and greatly increased the expression of genes involved in differentiation	[94]
HAp	Reduced Graphene Oxide	hMSCs	rGO - coated HA not only significantly enhanced osteogenic differentiation capacity of hMSC in osteogenic medium, but also significantly increased differentiation ability in basal medium.	[51]
GO-PEDOT	Reduced Graphene Oxide	hBMMSCs	GO-PEDOT controlled osteogenic differentiation of hMSCs through electrical stimulation.	[99]

*hBMMSC* human bone-marrow mesenchymal stem cell, *GF* graphene foam, *GC* graphene/calcium silicate, *HAp* hydroxlyapatite, *PEDOT* poly (3, 4-ethylenedioxyphene)

**Fig. 7:**
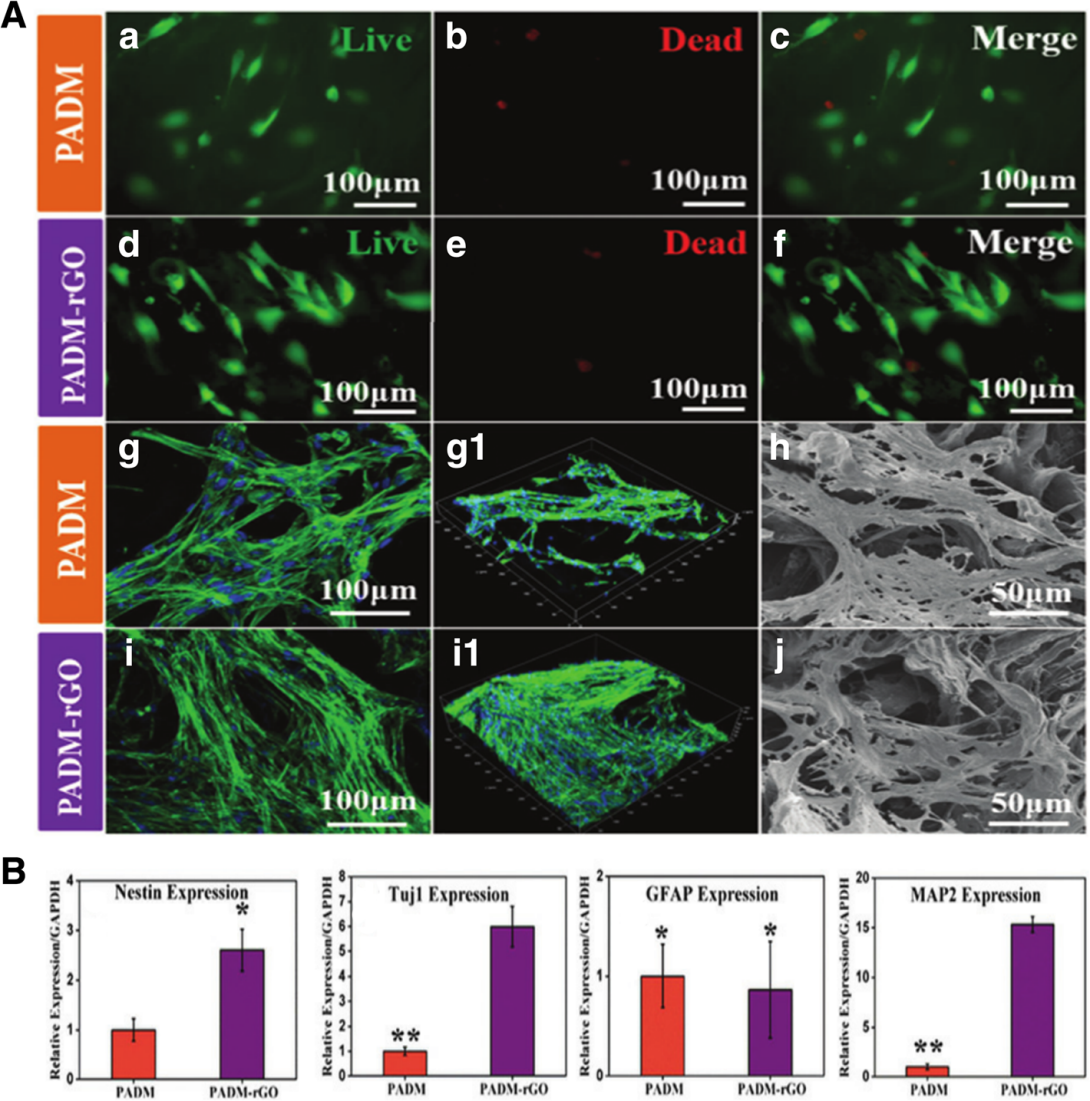
The effects of 3D porcine acellular dermal matrix (PADM) and PADM-reduced graphene oxide (PADM-rGO) on the adhesion and neuronal differentiation of human mesenchymal stem cells (hMSCs). (**a**) The cytocompatibilities of the two different scaffolds. The hMSCs were cultured on the PADM (a, b, c) and PADM–rGO (d, e, f) for 24 h, Live/dead staining was performed. The live cells are stained green, and dead cells are red. CLSM fluorescence morphologies of the actin cytoskeleton of the hMSCs cultured on the PADM (g) and PADM–rGO (i) scaffolds for 3 days. (h – j) SEM images represent the cell attachment of hMSCs after 3 days on the PADM and PADM-rGO. (**b**) Quantification of qPCR analysis for neural marker genes; Nestin, Tuj1, GFAP, and MAP2, expression of hMSCs. Copyright © 2015, Royal Society of Chemistry

Guo et al. suggested a novel 3D scaffold for neural differentiation of hMSCs. They used a 3D porcine acellular dermal matrix (PADM), mostly comprised of collagen I as a base scaffold, and assembled a layer of reduced graphene oxide (rGO). The fabricated PADM-rGO demonstrated an effective electrical conductivity and a typical porous structure (pores ranging from 50 to 150 μm in size). The hMSCs were then cultured on PADM and PADM-rGO for 24 h and underwent live/dead cellular staining. The cells maintained the archetypal spindle shape of hMSCs as seen in Fig. 7A a-f [9, 87, 88]. After 3 days of cultivation on each scaffold, the immunofluorescence images indicated that the density of cells on PADM-rGO was slightly higher than that of the cells on PADM (Fig. 7A g-i). As shown in Fig. 7B, the neural specific gene expression of cultured hMSCs for 7 days demonstrated that PADM-rGO accelerated the differentiation of hMSCs into neural cells.

Among numerous candidates, it has been previously studied that composite coating with HA/CNTs presented higher durability and longer maintenance period than the conventional HA coating [89–91]. In this regard, graphene has received substantial attention, which is composed of the same material as CNTs [92], but has a higher surface area, thermal conductivity, and flexibility. It is also well known for its high biocompatibility and harmlessness, which is considered to be important for grafting [93]. In this regards, Xie et al. studied graphene-reinforced calcium silicate coating (GC) technique, which was found be effective to generate a hierarchical nano−/microstructured surface [94]. The hMSCs were cultured on the GC. As a result, the wear resistance was increased compared with the conventional CS coating, and the adhesion and proliferation of hMSCs in vitro were enhanced when the GC coating was applied. In addition, it was confirmed that gene expression related to osteogenesis, alkaline phosphatase (ALP), osteocalcin (OC), and osteopontin (OPN), was increased. In addition, rGO exhibits exceptional properties, similar to properties of graphene, as mentioned above. In addition, it has been recently shown by many researchers that graphene has the potential to guide osteogenesis of hMSCs [4, 11]. Hydroxylapatite (HA), in the form of microparticles, forms a three-dimensional environment to enhance cell adhesion and proliferation [95]. By employing the advantages of these two materials, the authors developed rGO coated HA. Moreover, they demonstrated the enhancement of osteogenic differentiation of hMSCs when incubated in basal medium without any osteo-inductive molecules [51]. In addition, the osteogenic activity of cells was further improved in osteogenic medium. The researchers assumed that the initial exposure of rGO-coated HA to cells facilitated intracellular signaling via a more intricate pathway. However, further research is required to explore the actual mechanism.

**Fig. 8:**
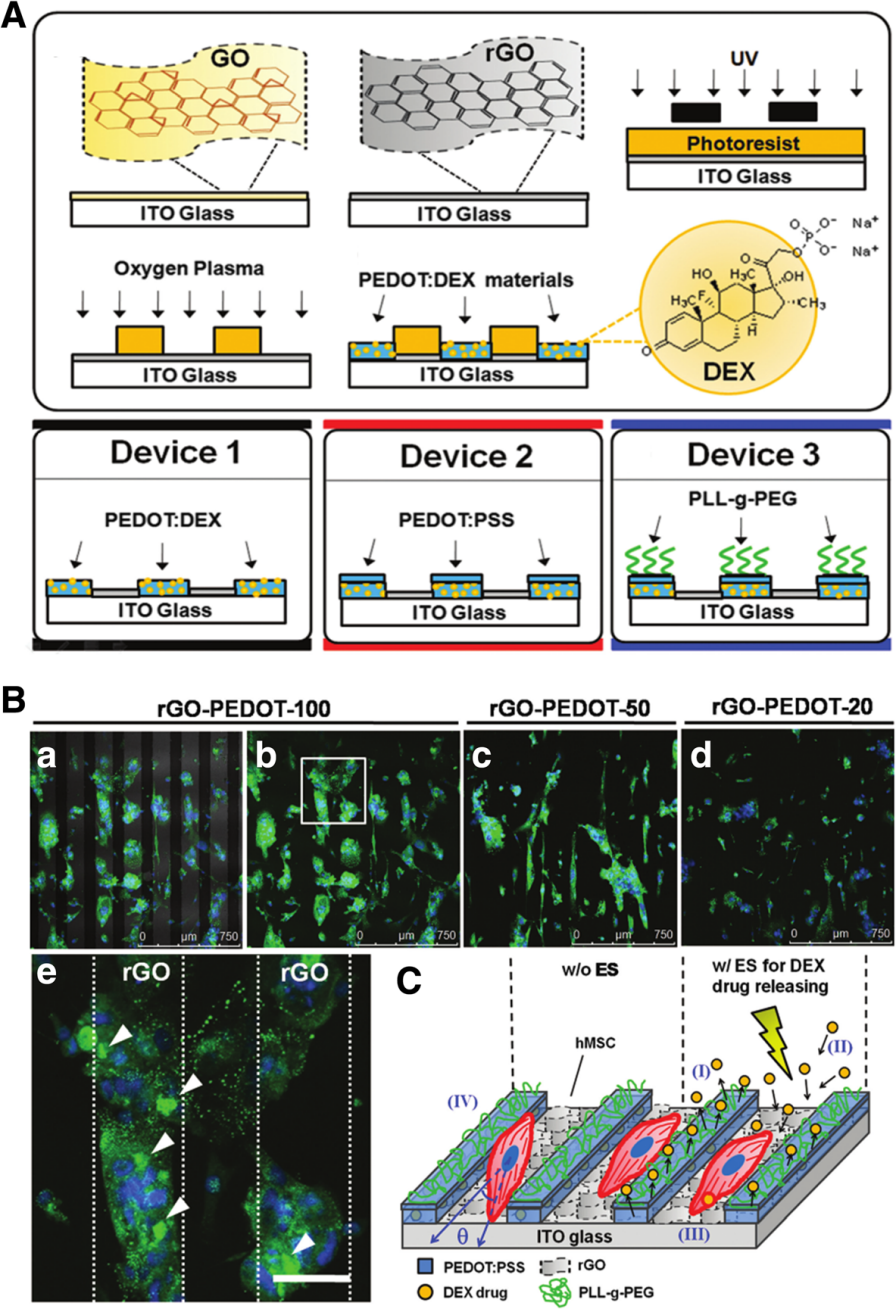
The osteogenic differentiation of human mesenchymal stem cells (hMSCs) induced by electrical release of differentiation factor, dexamethasone (DEX). (**a**) Schematic diagram represents the fabrication process of DEX-loaded bioelectrode array. (**b**) Immunofluorescence images of OCN expression in hMSCs cultured on various sizes of rGO-PEDOT (rGO-PEDOT-20, rGO-PEDOT-50, and rGO-PEDOT-100). (**c**) Schematic representation for rGO-PEDOT behavior. Copyright © 2013 WILEY-VCH Verlag GmbH & Co. kGaA, Weinheim

While scientists have traditionally relied on physical or chemical methods to examine biological entities, certain biological information (gene expression, differentiation, proliferation) can be obtained and monitored using electrical stimulation [96–98]. Hsiao et al. designed a novel 3D cell culture electrode with multifunctional graphene-PEDOT microelectrode and successfully controlled the osteogenic differentiation of hBMMSCs through electrical stimulation [99]. Materials used in this platform were electrically conductive indium tin oxide (ITO) glass and poly (3, 4-ethylenedioxyphene) (PEDOT) [100]. In addition, they used reduced graphene oxide (rGO), which is known to promote differentiation of hMSCs, to promote cell adhesion [95]. As a result, the researchers created the platform illustrated in Fig. 8a. The PEDOT pattern containing dexamethasone 21-phosphate disodium (DEX), which is one of differentiation inducers required for hMSC osteogenesis, and the hMSC aligned between them are cultured under the influence of the rGO. Subsequently, the PEDOT released the DEX only when an electric stimulation was provided (Fig. 8b). Therefore, Hsiao’s platform is shown to easily control the differentiation of hMSCs using only the electrical stimulation.

Conclusively, we have highlighted several 3D graphene-based platforms as a substrate for differentiation of hMSCs in this Review. The biocompatibility of these modified 3D scaffolds could be widely utilized for tissue engineering applications such as bone regeneration therapy.

## Conclusion

In this review, we focused on several studies that used various nanohybrid materials for biomedical applications, with a particular focus on the use of two-dimensional materials, gold nanoparticles, and three-dimensional graphene composites [101].

Some of the prior reports have confirmed that two-dimensional materials and nanomaterials in combination with biological materials (e.g., growth factors, peptide, and proteins) enhance a number of cellular behaviors including cell adhesion, proliferation, migration and differentiation. Interestingly, these materials were especially excellent in performing as an attracting signal, not just for the osteogenesis of hMSCs but also for the enhancement of bone regeneration process.

In addition, three-dimensional carbon nanomaterials also have been utilized as the platform to support stem cell growth and differentiation. Unlike the two-dimensional platforms, which turned out to be suitable for controlling stem cells functions/behaviors in vitro, the three-dimensional carbon nanomaterials were found to be excellent in constructing 3D in vivo-like conditions ex vivo. Such approaches were useful to mimic structures of human tissues/organs, which is critical for the development of a new types of in vitro drug-screening tool such as organ-on-a-chip, as well as to develop tissue-biomaterial composites for the transplantation purpose. Although the use of bionano platforms for tissue regeneration is still in its early stages of development, certain biochemical and physical properties of those platforms, which includes tunable physical sizes, shapes, surface hydrophilicity, functional groups, entail a promising future for its development in biomedical fields, especially for the stem cell-based regenerative therapies.

## Abbreviations

ARS: Alizarin red S
AuNPs: Gold nanoparticles
CNT: Carbon nanotube
CS: Calcium silicate
CVD: Chemical vapor deposition
ECM: Extracellular matrix
GO: Graphene oxide
HA: Hydroxylapatite
hMSCs: Human mesenchymal stem cell
